# Development and validation of a comprehensive patient‐specific quality assurance program for a novel stereotactic radiation delivery system for breast lesions

**DOI:** 10.1002/acm2.12778

**Published:** 2019-12-13

**Authors:** Stewart J. Becker, Ying Niu, Yildirim Mutaf, Shifeng Chen, Yannick Poirier, Elizabeth M. Nichols, ByongYong Yi

**Affiliations:** ^1^ Department of Radiation Oncology University of Maryland School of Medicine Baltimore MD USA; ^2^ MedStar Georgetown University Hospital Washington DC USA

**Keywords:** breast, commissioning, patient specific quality assurance, stereotactic radiosurgery

## Abstract

**Purpose:**

The GammaPod is a dedicated prone breast stereotactic radiosurgery (SRS) machine composed of 25 cobalt‐60 sources which rotate around the breast to create highly conformal dose distributions for boosts, partial‐breast irradiation, or neo‐adjuvant SRS. We describe the development and validation of a patient‐specific quality assurance (PSQA) system for the GammaPod.

**Methods:**

We present two PSQA methods: measurement based and calculation based PSQA. The measurements are performed with a combination of absolute and relative dose measurements. Absolute dosimetry is performed in a single point using a 0.053‐cc pinpoint ionization chamber in the center of a polymethylmethacrylate (PMMA) breast phantom and a water‐filled breast cup. Relative dose distributions are verified with EBT3 film in the PMMA phantom. The calculation‐based method verifies point doses with a novel semi‐empirical independent‐calculation software.

**Results:**

The average (± standard deviation) breast and target sizes were 1263 ± 335.3 cc and 66.9 ± 29.9 cc, respectively. All ion chamber measurements performed in water and the PMMA phantom agreed with the treatment planning system (TPS) within 2.7%, with average (max) difference of –1.3% (−1.9%) and −1.3% (−2.7%), respectively. Relative dose distributions measured by film showed an average gamma pass rate of 97.0 ± 3.2 when using a 3%/1 mm criteria. The lowest gamma analysis pass rate was 90.0%. The independent calculation software had average agreements (max) with the patient and QA plan calculation of 0.2% (2.2%) and −0.1% (2.0%), respectively.

**Conclusion:**

We successfully implemented the first GammaPod PSQA program. These results show that the GammaPod can be used to calculate and deliver the predicted dose precisely and accurately. For routine PSQA performed prior to treatments, the independent calculation is recommended as it verifies the accuracy of the planned dose without increasing the risk of losing vacuum due to prolonged waiting times.

## 1 INTRODUCTION

The GammaPod (Xcision Medical Systems, LLC; Columbia, MD) is a novel breast‐specific stereotactic radiotherapy device developed at the University of Maryland that has recently received 510(k) clearance from the U.S. Food and Drug Administration (FDA).^1^ Operating similarly to the long‐established Gamma Knife, the GammaPod’s 25 nonoverlapping cobalt‐60 (^60^Co) sources dynamically paint dose to a breast lesion by rotating the beams around a small target while the couch translates continuously in three axes during beam delivery. From simulation to treatment, the patient’s breast is immobilized using mild negative pressure through a device‐specific dual‐cup system with stereotactic fiducials. The cup, which functions as a stereotactic frame, is secured to both the simulation and treatment tables, ensuring a single stereotactic set of coordinates for fast and accurate localization. A novel treatment planning system (TPS) optimizes planned dose distributions by varying the collimator size (15 and 25 mm), isocenter position, and beam‐on time for each specific isocenter position. TPS calculations are derived from Monte Carlo calculations performed in water with a breast density of 0.935 g/cm^3^. Details of the GammaPod and the GammaPod TPS can be found in a previously published reports.^1^, ^2^, ^3^

Whenever a large stereotactic dose is delivered to a small volume, geometric misses and errors in delivery can have a large impact on the resulting quality of treatment.^4^, ^5^, ^6^ Indeed, studies have shown the importance of independent pretreatment verification with similar modalities, such as the GammaKnife.^7^, ^8^ The objective of this study is to develop and report a comprehensive patient‐specific quality assurance (PSQA) program for the GammaPod. To this end, we perform absolute ionization chamber measurements and relative film measurements, which we then compare to predicted TPS dose distributions for the initial 15‐patient cohort of our trial.

This study represents the first validation of the ability of the novel GammaPod dedicated breast stereotactic treatment unit to accurately plan and deliver stereotactic radiosurgery (SRS)–grade radiation dose distributions. It also represents the first PSQA performed using the patient geometry with the actual patient’s plan.

## 2 METHODS

### 2.1 The GammaPod breast stereotactic radiosurgery (SRS) system

The GammaPod design was originally described by Yu et al.^1^ It includes three main systems: the collimator and source carrier, the couch, and the breast cup. The accuracy of the GammaPod system has been reported in Becker et al.^3^ The GammaPod’s collimator produces and beam with a FWHM that is reproducible to within ≤ 0.2 mm, the couch translation is reproducible within ≤ 0.1 mm, and the registration of the breast cup fiducial system is accurate within ≤ 0.2 mm.

#### 2.1.1 Collimator and source carrier

**Figure 1:**
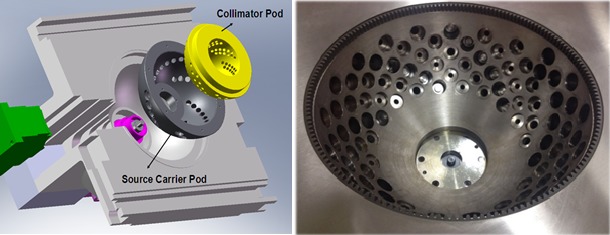
A diagram of the source carrier and collimator system.

**Figure 2:**
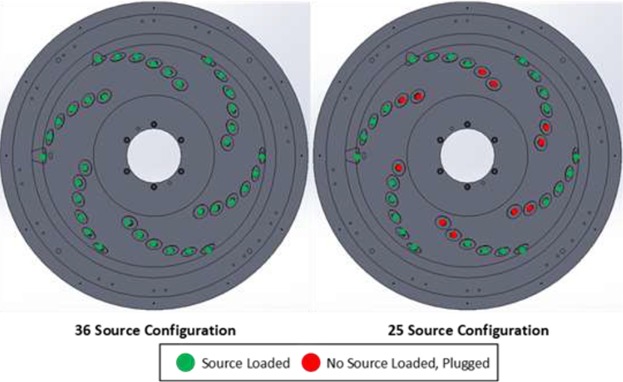
Top‐down view of the collimator system showing the source distributions for both the 36‐ and 25‐source models.

The collimator and source carrier include two independent hemispheres that are nested together. The outer hemisphere is the source carrier, which houses the 25 (previously 36 in an earlier design^1^, ^2^) sources which are aligned in a spiral pattern around the carrier, all focused at an isocenter 380 mm away. Starting at 18° below the horizontal plane, the sources spiral down by 1° off the horizon and 60° longitudinally at a time. In contrast, the inner hemisphere plays the role of the collimator. This inner hemisphere holds openings for both 15‐ and 25‐mm diameter field sizes which can align with each of the ^60^Co sources, selecting among them through a 20° rotation between the “blocked” and 15‐ and 25‐mm positions. To deliver treatment, the entire assembly rotates as a single unit to spread the dose from all the sources around the breast. This geometry is illustrated in Fig. 1, a diagram of the source carrier and collimator system. Fig. 2 is a top‐down view of the source configuration for both the 36‐ and 25‐source models.

#### 2.1.2 Patient couch

The second component of the GammaPod system is the patient couch. This couch system holds and immobilizes the patient and locks her into place via the breast cup. The table lowers the patient into position so that the radiation isocenter is within the breast & PTV. The couch then translates the patient along the planned path so that the target receives the desired dose distribution. This is quite similar conceptually to the use of multiple isocenters in the GammaKnife, with the exception that the GammaPod table moves continuously between tens to hundreds of isocenters during treatment, while GammaKnife delivers radiation only in discrete isocenters. This dynamic delivery method enables a much more uniform dose distribution inside the target compared to the “sphere packing” technique characteristic of the Gamma Knife.^9^, ^10^

#### 2.1.3 Breast cup system

**Figure 3:**
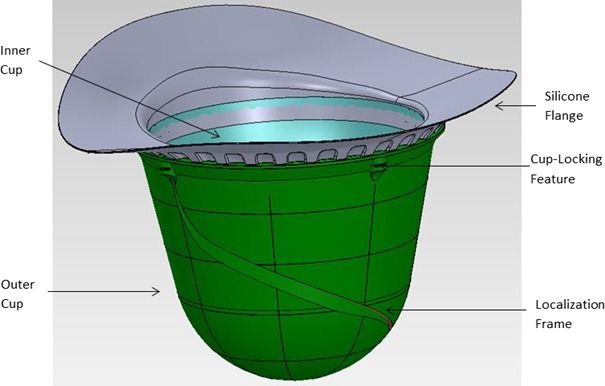
Diagram of the cup system.

**Figure 4:**
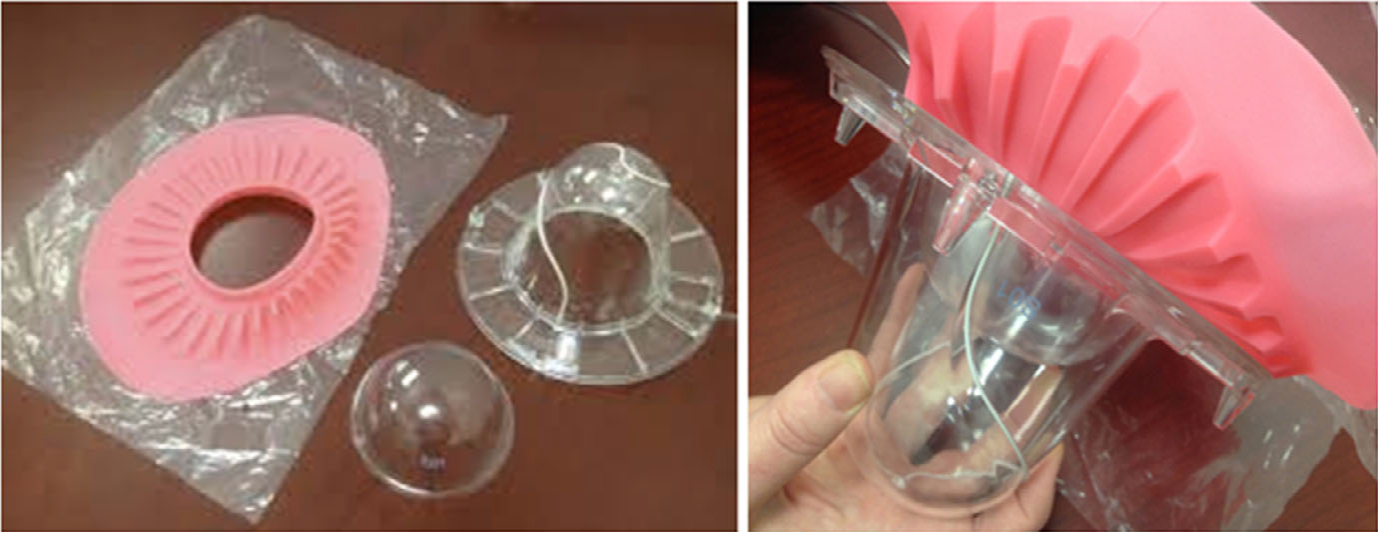
(Left) a photo of the pink flange, inner cup, and outer cup. (Right) the all three pieces assembled.

The third and final component of the GammaPod is the breast cup system, which serves as an immobilization device and provides the stereotactic frame. It consists of an outer cup, an inner cup, and a flange (Fig. 3). Outer cups are in three diameters (small, medium, and large) and are made of a hard and rigid polycarbonate. The small, medium, and large sizes correspond to diameters at the base of 93.7, 121.7, and 153.7 mm, respectively. This outer cup locks to the table to keep the patient immobilized. Embedded in the cup is a fiducial wire (recognized by the TPS) that serves to determine the laterality of the treatment and the stereotactic coordinate reference frame. The outer cup is hermetically sealed, except for a tube that connects to the vacuum pump. The inner cup, constructed with a thin layer of polyethylene, is available in the same three diameters as the outer cups and in 10 sizes for each diameter, based on chest‐to‐apex distances (Fig. 4). This inner cup is selected based on the patients’ breast size so that the entire cup is filled, except for a small air gap at the apex of the breast, ensuring a predictable treatment geometry for planning purposes. A seal between the inner cup and the patient’s skin is achieved using a silicone flange fitted to the inner cup. The flange is then attached to the outer cup. When the vacuum is applied between the outer and inner cups, aeration holes close to the apex of the breast produce suction that immobilizes the breast and the chest wall for simulation and treatment delivery.

### 2.2 Original clinical trial

This study was conducting using the planning and measurement information of 15 patients who underwent GammaPod treatments under the IRB approved protocol to collect safety and feasibility data in preparation for submission for FDA 510(k) clearance.^11^ These 15 original patients were treated with a single‐fraction 8‐Gy boost to the lumpectomy cavity + a 1‐cm margin.^12^ After GammaPod treatment, patients proceeded to receive whole‐breast radiotherapy using a hypofractionated course of therapy consisting of 40.05 Gy in 15 fractions or 42.56 Gy in 16 fractions. Table 1 displays the target and plan details for each patient.

**Table 1:** Plan attributes of the 15 patients.

Patient #	GTV Vol. (cc)	PTV Vol. (cc)	Normal Breast Vol. (cc)	Control Points	Location in Breast	Proximity
1	3.8	21.7	1005	371	Upper‐Outer	CW
2	20.4	89.6	1463	361	Upper‐Outer	skin, CW
3	2.9	32.5	722	813	Middle‐Outer	skin
4	9.7	65.6	1553	463	Middle	
5	13.5	74.9	800	426	Middle	
6	11.3	62.5	1994	383	Middle	skin
7	6.1	45.2	1384	445	Upper‐Outer	CW
8	6.4	61.9	1314	510	Middle	CW
9	6.6	53.4	1554	414	Lower‐Outer	skin
10	5.3	45.7	1264	456	Middle	
11	15.5	89.9	1463	472	Upper‐Outer	skin
12	12.7	78.8	727	424	Middle	
13	29.1	153.9	1461	736	Lower	
14	16.7	87.0	1309	648	Middle	
15	6.2	40.6	949	440	Outer‐Lower	skin
min	2.9	21.7	722	361		
max	29.1	153.9	1994	813		
avg	11.1	66.9	1264	491		

GTV, gross tumor volume; PTV, planning treatment volume.

### 2.3 Patient‐specific QA program

We validated the ability of the GammaPod SRS device to accurately and precisely deliver conformal high‐dose distributions. Three components were employed: absolute ionization chamber measurements, relative film measurements, and an independent calculation program.

#### 2.3.1 Absolute measurements in water

We measured the absolute dose using the TG‐21 formalism under two geometries: a water cup matching the patient, and a polymethylmethacrylate (PMMA) semicylindrical phantom. In both cases, measurements were performed using an Exradin A1SL (0.053 cc) thimble chamber and a CDX 2000B Electrometer (both from Standard Imaging, WI). The chamber was chosen since it was made specifically for small field sizes that are utilized by the machine. The full width half‐maximum for the smallest collimator is ~22.0 mm.

**Figure 5:**
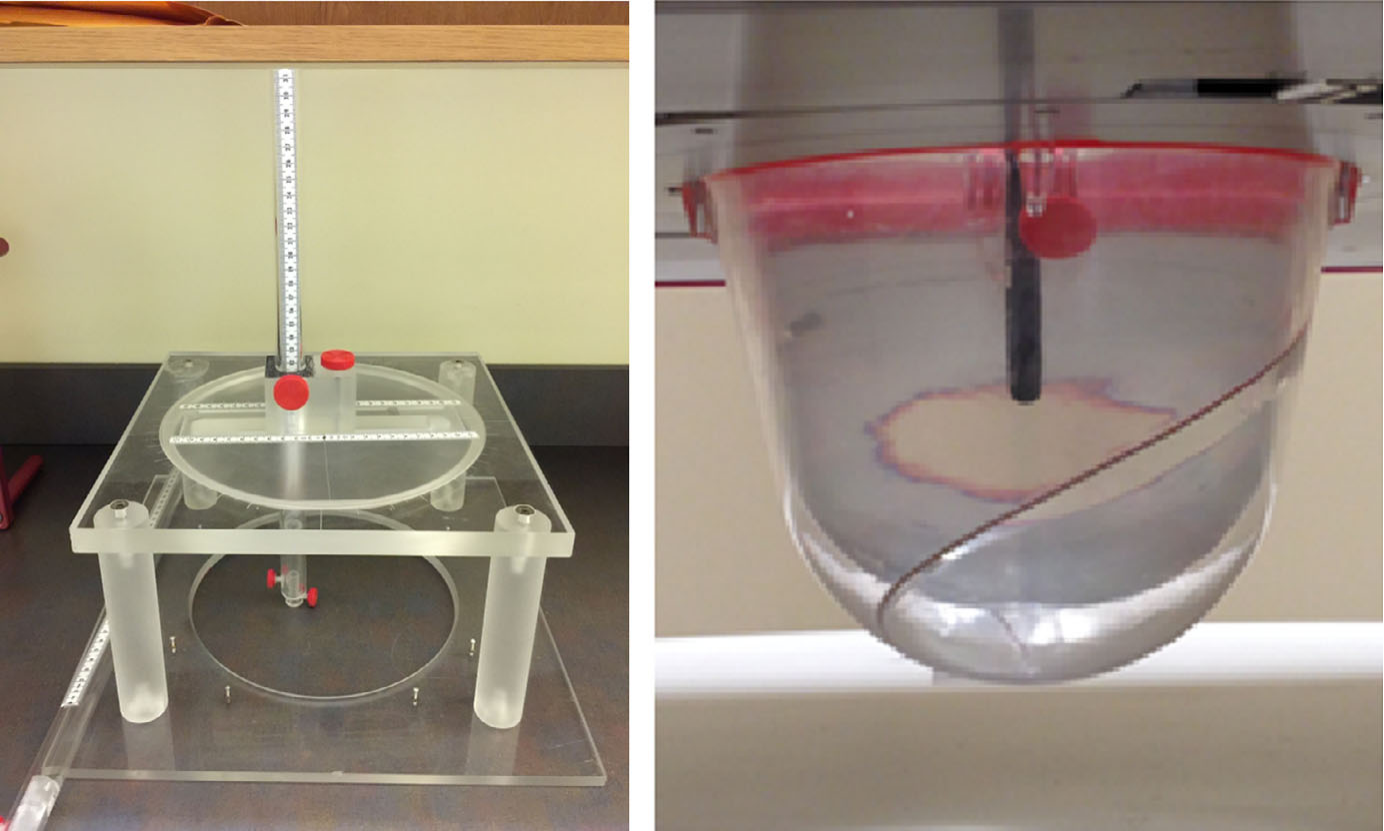
Jig designed to position an ion chamber for measurements in the water‐filled breast cup. The jig is designed so that the ion chamber can move along two linear axes, as well as rotate, allowing the ion chamber to be precisely placed at any point within the breast cup to match the location of the tumor within the breast in the patient‐specific geometry.

The first measurement geometry (e.g., the water cup) was chosen to utilize the specific cup used to immobilize and treat the patient. To prevent spills, ventilation holes located at the tip of the cup are taped closed (typically under suction to immobilize the breast), and the inner cup is filled with water. We designed and fabricated a custom jig to sit atop the couch over the water‐filled cup and which locks in with pins to position the ion chamber precisely and reproducibly. This jig allows the ion chamber to reach all positions in the breast. In the TPS, a point is picked within the gross tumor volume (GTV) at which the dose distribution exhibited a relatively low‐gradient region (<0.5% over 1 mm), and its spatial coordinates (expressed as X, Y, Z) and dose are recorded. The TPS displays the dose from a single pixel, 0.001 cc and also the average dose from 5 × 5 × 5 pixel matrix around the center pixel, 0.125 cc. This averaging of the point dose over the surrounding pixels creates a volume closer to the volume of the ionization chamber utilized, 0.125 cc (5 × 5 × 5 average) vs. 0.053 cc (chamber) vs. 0.001 cc (single pixel of TPS). The coordinates are then converted to the jig cylindrical coordinate system such that the ion chamber can be placed at that same point in the cup. This setup is shown in Fig. 5. The calculated doses are then compared to the measurements. To correct for the density difference between the water measurements and the breast density water tissue used by the TPS, an average density correction factor of 1.6% was utilized. This factor was derived by Monte Carlo calculations to take into account the average attenuation difference between the two mediums.

#### 2.3.2 Absolute measurements in the PMMA phantom

**Figure 6:**
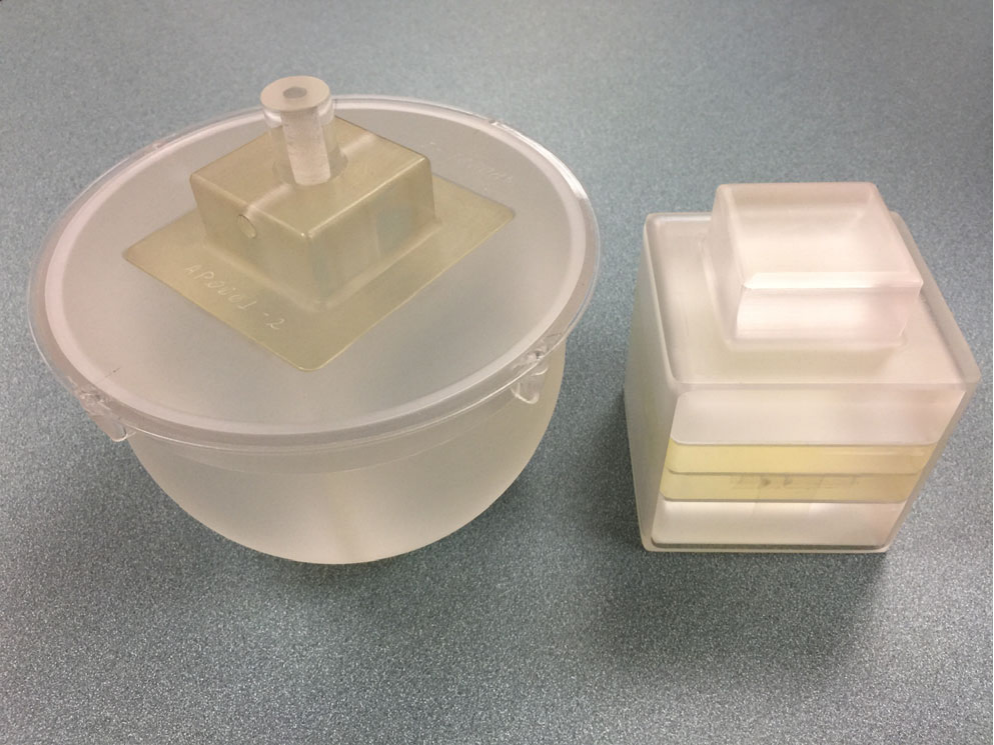
PMMA breast phantom with ion chamber installed (left) and film insert (right). The PMMA phantom can accommodate either insert with no effect on the measured dose distribution. The film holder is capable of measuring 2D dose distributions in three planes simultaneously and can be rotated in three perpendicular orientations for greater flexibility. PMMA, Polymethylmethacrylate.

We also performed absolute dose measurements in a PMMA phantom using the same A1SL chamber. The PMMA phantom was supplied by Xcision Medical Systems and includes a cavity capable of accommodating either an ionization chamber or film (Fig. 6). The reason for measuring the absolute dose in the PMMA phantom is to validate the absolute dose in the same geometry used to perform the relative film measurements (see section 2.3.3). In contrast, the water phantom measurements described in section 2.3.1 are more representative of the absolute dose delivered to the patient, because the ionization chamber can be placed in a position representative of the target volume in a cup of the same size as that used to treat the patient.

The TPS automatically creates a QA plan in which the patient’s plan is recalculated on the PMMA QA phantom. However, the PMMA phantom accommodates only a central location for its ionization chamber, whereas in the water cup the dose distribution remained centered on the position of the GTV. To avoid missing the target or dose–volume averaging effects caused by measuring dose in a high‐gradient region, it is therefore necessary to center the planned dose distribution on the ion chamber. This is achieved by shifting the centroid of the planning treatment volume (PTV) to the sensitive volume of the ion chamber. The calculated dose is then directly compared to the measurement. All other parameters of the plan remain the same (i.e., collimator size and time at each position). Only the table (x, y, z) positions are adjusted to move the centroid of the GTV to the ion chamber location.

#### 2.3.3 Relative dose distributions measured by Gafchromic film

**Figure 7:**
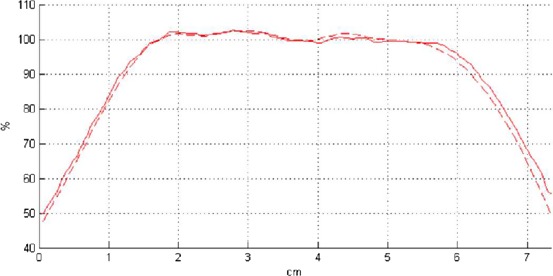
Comparison of measured (full line) and predicted (dashed line) dose profiles for a representative patient. In general, the dose agrees within < 1 mm or 1%.

The PMMA phantom described in the previous section is also capable of accommodating film. This is accomplished by removing the ion chamber insert and replacing it with the film holder. Using EBT3 Gafchromic (Ashland Inc., Wayne, NJ) film placed in the XZ plane, we measured relative dose distributions and compared these to those predicted by the TPS (Fig. 7). These measurements utilize the same QA plan created in the subsection above for the absolute point dose measurements in the same phantom.

The film was previously calibrated using a 6‐MV X‐ray beam with 12 dose points between 0.01 and 10 Gy. All films were cut and scanned using standard Gafchromic film procedures (i.e., using the red channel only).^13^ The film holder has four pins to mark the film for registration. When a QA plan is generated from a patient’s plan, four marks are burned to dose planes to allow for image registration with the film. Fig. 7 shows the comparison between a film and calculated dose profile. The film dose was normalized to a plateau region in the target and analyzed with 3%/1 mm gamma criteria. This criteria is the standard for SRS PSQA.^13^

#### 2.3.4 Independent point dose calculation software

Our third PSQA component is a semi‐empirical independent point dose calculation (SEIPDC) to verify the integrity of the TPS calculation. The calculation revolves around using a kernel approach, utilizing predefined isocenter dose rates and off‐center ratios (OCRs). The isocenter dose rates are the dose rates at isocenter for each possible position in the breast. If the isocenter is positioned near the chestwall of a large breast, the dose rate will be less than when the isocenter is positioned at the apex of a smaller breast. The predefined OCR is calculated from the dose distribution of each cup when centered at the isocenter position. Both the isocenter doses and OCRs come from a Monte Carlo engine that has been pre‐calculated on each of the devices 19 inner cup sizes. Each plan consists of a series of control points which consists of a table coordinates (*x,y,z*), colliminator size, and time at that location. The first‐order approximation of the dose contribution from each control point to the reference point is achieved by overlaying all shot locations and summing the individual dose contributions from each control point (i = 1:n) to the reference point according to the formula:

(1) $$D_{\text{ref},\;\text{no volume}}=\left[\sum_{i=1}^{n}D_{i}\left(C_{i},c_{i},y_{i},r_{i,}t_{i}\right)*{\text{OCR}}_{i}\left(C_{i},y_{i}-y_{\mathrm{ref}},r_{i}-r_{\mathrm{ref}}\right)\right],$$

where *D*_i_ represents the isocenter dose at center of control point (i) which itself is dependent on collimator size [*C*], cup size [*c*], the Y‐position [*y*], the distance from the central axis [*r*] and dwell time [*t*]), OCR*_i_* is dimensionless and represents the relative off‐center ratio at a distance r from control point (i), and *D*_ref, no volume_ represents the total dose to the calculation reference point which does not consider the volume effect. Eq. (1) uses the OCR of the profile of the centrally located beam for approximation. Eq. (1) is valid only if the dose distribution of each collimator is positionally invariant. However, the dose distribution is distorted unless the isocenter is located at the center of the cup. A correction is needed if a control point is not located at the cup center, which happens all the time when the control points are more than one and happens even more frequently when the target volume is larger. The correction factor $V_{f}$, is empirically determined through a relationship between the volume treated and the difference between the calculations using the Eq. (1) and the measurements. It is also a dimensionless factor. Then dose to the reference point can be acquired as following;

(2) $$D_{\mathrm{ref}}=\left[\sum_{i=1}^{n}D_{i}\left(C_{i},c_{i},y_{i},r_{i,}t_{i}\right)*{\text{OCR}}_{i}\left(C_{i},y_{i}-y_{\mathrm{ref}},r_{i}-r_{\mathrm{ref}}\right)\right]V_{f},$$

$V_{f}$ is a volume dependency correction factor which is used to correct for the distortion of the dose distribution when it is off from the center of the cup.

We use Eq. (2) to calculate the dose to a reference point in the PMMA phantom and to the patient. These calculated point doses are then compared to the doses calculated by the TPS in the PMMA phantom and the water cup. The dose calculation to the PMMA phantom verifies the accuracy of the dose distribution to a known geometry, and the point calculation in the water cup verifies the dose in the patient‐specific geometry.

## 3 RESULTS

We developed PSQA tests to verify planned dose distributions in the initial 15‐patient study. These patients presented with a range of breast (and thus, cup) and tumor sizes, averaging 1263 ± 335.3 cc and 66.9 ± 29.9 cc, respectively.

**Figure 8:**
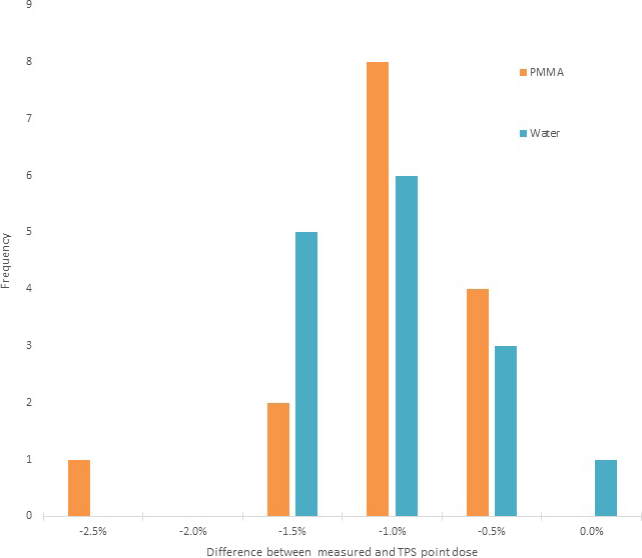
Histogram of distribution of the percent difference between point dose measurements and TPS calculation in the PMMA phantom and the patient‐specific water‐filled breast cups for all 15 patients of the initial clinical study. PMMA, Polymethylmethacrylate.

**Figure 9:**
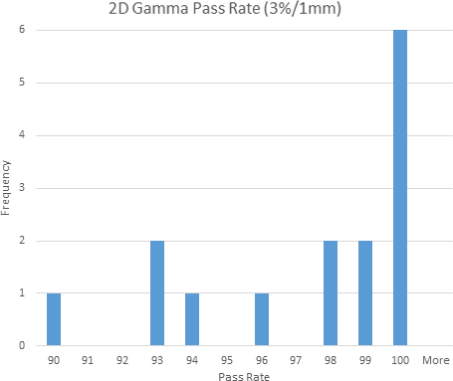
Histogram of the distribution of the gamma index between profile measurements and the TPS calculation in the PMMA phantom for the 15 patients from the initial clinical study. PMMA, Polymethylmethacrylate.

Ion chamber measurements in water and in PMMA had an average agreement to those of the TPS of −1.3 ± 1.0% and −1.3 ± 0.5%, respectively. The largest outliers for each were −1.9% and −2.7% respectively. The profile measurements had an average gamma pass rate of 97.0 ± 3.2% when using 3%/1 mm, with a worst agreement of 90.0% for one patient. The independent calculation software had an average agreement with the water‐based calculations and the PMMA QA calculations of 0.2 ± 1.2% and −0.1 ± 1.1%, respectively. These results are shown in Table 2 and Figs. 8 and 9.

**Table 2:** Measurement and independent point dose calculation results averaged over all 15 patients.

Phantom	Measured vs TPS avg (max, min)	SEIPDC vs TPS avg (max, min)
PMMA	−1.3% (−0.5%, −2.7%)	−0.1% (1.5%, −2.0%)
Water	−1.3% (0.0%, −1.9%)	0.2% (2.2%, −2.0%)

PMMA, Polymethylmethacrylate; TPS, treatment planning system.

## 4 DISCUSSION

This study represents the first experimental verification of the GammaPod device’s ability to accurately and precisely calculate and deliver highly conformal SRS doses to the prone breast. While three examples of radiation treatment plans created by the GammaPod TPS had been presented as part of the original publication describing the device’s theoretical design,^1^ these plans had been entirely theoretical and had not been based on patient data, nor had their delivery feasibility or accuracy been verified. We developed a PSQA method for our initial 15‐patient study that showed excellent agreement between the TPS and the point dose and film measurements. As seen in Figs. 8 and 9, absolute point dose measurements agreed with TPS calculations within ≤2% for each patient in both the PMMA and the water cup, except for a single 2.7% outlier in one case.

**Figure 10:**
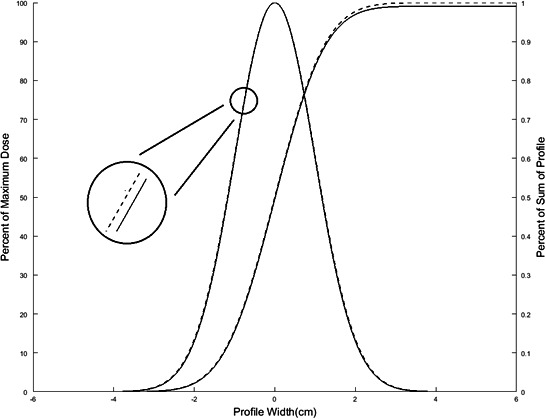
Two theoretical 15‐mm profiles overlaid. Profile widths differ by 0.2mm in total width and the area Table 1: Plan attributes of the 15 patientsunder the two profiles which differ by 1%.

Of particular note, the point dose measurements, both in the PMMA phantom and water‐filled breast cup, were consistently below TPS calculations. We believe the cause is related to small uncertainties in the collimator openings which lead to a narrowing of the profile width in a process analogous to the dynamic leaf gap (DLG) commonly used in intensity‐modulated radiation therapy. In the DLG, a small error in field size (i.e., in the gap between two multi‐leaf collimator leaves) has a negligible effect on the output factor, but can have a disproportionate effect on the total absolute dose delivered by an IMRT plan with a sliding‐window delivery. This is due to the fact that the gap is scanned across the target in many occasions, and that dose to a point is related to the time it is “seen” by the MLC gap, which increases when the DLG is larger. Likewise for the GammaPod, the planned dose distribution is delivered through a series of isocenters, and radiation delivery continues in‐transit while the patient is moved between isocenters. Therefore, the absolute dose is related to the total area under the curve of a radiation profile. A simplistic approximation shows that, for a 15 mm collimator, an error of 0.2 mm (which would be very challenging to measure experimentally) will cause a difference of 1% in area under the curve, and the absolute dose difference will depend on the final isocenter placement and the particular parameters of the plan. Fig. 10 shows two theoretical profiles with a 0.2mm width difference and it also displays the sum under the curve which represents total dose collected from scanning across the profile. As one can see the 0.2mm difference is not visible but the 1% difference in sum is. This does not affect the output calibration of the machine since the measurement is performed with a static beam centered on the isocenter. We are currently investigating various methods by which to empirically detect this possible cause of the discrepancy between planned and delivered dose.

One of the novel aspects of this study is that the use of a water cup represents one of the first uses of the exact patient geometry — along with the specific patient plan — to measure the dose delivered to the patient. It does not involve using another, non‐representative phantom geometry, or even a recalculation of the dose. For this reason, our study is able to detect the real impact on the absolute dose delivered to the patient arising from such uncertainties in a way that calculation or non‐representative phantoms cannot.

The use of a QA phantom brings up the same dilemma that all other IMRT or VMAT QA experiences that how does one reconcile using a phantom that is not the patient to verify a dose to an actual patient. While QA treatment is technically a different plan it is essentially the same plan meaning it uses the same beam on times, collimator sizes, and the same table motion. The only difference is that the absolute position of the table is different. This is the same for IMRT and VMAT QA where the gantry speed, gantry angles, MLC positions and MUs are the same only the couch is a different position because the phantom is not the same size as the patient.

The long‐serving Gamma Knife (Elekta; Stockholm, Sweden) represents the device closest in form and function to the GammaPod and served as inspiration in our development of QA procedures. This presented a challenge, because historically the Gamma Knife has been considered capable of delivering dose distributions accurately without the necessity of patient‐specific measurements. A number of reports have described PSQA for linac‐based SRS and SBRT.^13^, ^14^, ^15^, ^16^, ^17^, ^18^ In general, these articles described results similar to those we achieved with our PSQA program. Wen et al.^13^ using a 3%/1 mm gamma criteria achieved an overall pass rate of 95% for linac‐based SRS/SBRT. Kim et al.^16^ achieved an average gamma of 97.0 ± 2.5% for stereotactic ablative radiotherapy/volumetric‐modulated arc therapy (VMAT) with 2%/1‐mm criteria. Colodro et al.^14^ achieved an average gamma pass rate of 92.7 ± 2.9% with 3%/1.5‐mm gamma criteria for VMAT SBRT.

Several studies have previously reported on the development and use independent calculations,^7^, ^19^, ^20^, ^21^, ^22^, ^23^, ^24^, ^25^ as such we considered it prudent to develop a method of our own. Our SEIPDC software when compared to the TPS had an average agreement −0.1 ± 1.1% for the PMMA phantom and 0.2 ± 1.3% for the water phantom. These compare favorably to other independent calculation systems in the field. Marcu et al.^20^ and Zhang et al.^21^ both demonstrated algorithms with average agreements of ± 3% for multiple isocenter plans to the Gamma Knife planning system. Wright et al.^7^ reported agreement between their algorithm and Gamma Knife planning system of 0.3 ± 1.3%.

The SEIPDC software showed good matching to measurements and to TPS. Unlike other modalities, it is critical to shorten the waiting process. Performing measurement pretreatment may be desirable in principle, but it elongates the total waiting time, which increases the risk of losing vacuum. Considering the above practical reason and the calculation accuracy, the calculation‐based pretreatment PSQA may be an adequate method. The dimensionless volume correction factor, $V_{f}$, of Eq. (2) can be determined by comparing the volume and the calculation error of the Eq. (2) without the factor as it is described earlier. It is determined to be;

(3) $$V_{f=}0.028*v+2.7,$$

where, *v* is the volume of the target. Considering standard deviation (SD) of 1.4% between the phantom measurements and the calculation, more than 2.7 SD or less than 1% of the cases are expected to have larger than 3.8 % error to the measurements, which requires the measurement‐based PSQA before treatments. There were no Type I or Type II errors for the 3% passing criterion of the dose difference and 0.13 Type II error for the 2 mm criterion. Eq (2) provides no false pass for 3% or higher passing criterion.

Further development of the SEIPDC software towards 2‐D or 3‐D gamma comparison will even increase more confidence level.

Given the relatively few moving parts involved in the GammaPod system and its reliance on well‐characterized radioactive sources, catastrophic random errors in dose distribution are unlikely. As with Gamma Knife treatments, it is unclear that maintaining a long‐term PSQA will be necessary. However, when introducing a new technique in any center, it is vital to establish its feasibility across a wide variety of geometries and situations. Therefore, any new center implementing a GammaPod program could benefit from introducing a PSQA program such as that described in this study. This is further supported by recent trends toward failure modes and effects analysis of QA procedures, as have been outlined in publications based on the TG‐100 report.^26^, ^27^, ^28^, ^29^, ^30^, ^31^, ^32^

This study was conducted for 15 patients, which were scanned, simulated, planned and treated as part of the device’s initial trial submitted for FDA approval. While this is a limited number of patients, there is no consensus or guidelines on how many patients represent a sufficient number in order to validate a novel patient‐specific QA methodology and 15 patients compares favorably to similar studies. Indeed, Mamalui‐Hunter et al.^19^ used 15 patients to validate a QA methodology in the Gamma Knife, which is the most similar device currently in clinical use. Furthermore, the 15 patients chosen represents a wide variety of target sizes (20–150 cc), location (near the chest wall vs near the skin surface, central vs lateral), treatment complexities (300–800 control points), and breast volumes (700–2000 cc). Furthermore, seromas are always convex with spherical shapes as opposed to the often concave and complex target shapes in IMRT. Considering that lumpectomy cavity diameters tend to range from 0.5 cm to 4 cm^33^ with an additional 1‐cm PTV margin, we expect the PTV of most patients to fall within a 15 cc — 210 cc range. As such, the PTV volumes reported in this study are very representative of the expected patient population. In addition, unlike classic IMRT cases, patients treated on the GammaPod are always immobilized using the system’s breast cup system. These are limited in number and force the breast to adopt a specific shape, which means there is a very small number of possible external body geometries. In turn, this greatly limits the variety of dose distribution variations due to external contour variability. Finally, this study validates 8 of 20 possible outer/inner cup combinations (2 outer × 10 inner). While the absolute smallest and largest inner cups were not sampled due to lack of appropriate patients, a very wide range of possible geometries was still validated including the second smallest and largest inner cups.

## 5 CONCLUSION

We successfully implemented a PSQA program for the initial 15‐patient study used to obtain FDA 501(k) approval for the GammaPod SRS system. Using absolute ionization chamber measurements performed in water and in a PMMA phantom, as well as film relative‐dose measurements and an independent dose calculation, we validated not only the ability of the GammaPod to accurately deliver precise high‐dose distributions but also the ability of the GammaPod TPS to accurately plan and calculate these highly conformal treatment plans.

Considering the accuracy of the SEIPDC software and the clinical reason of avoiding the increased risk of losing vacuum, the calculation based PSQA is recommended. This study represents the first implementation of a PSQA for the GammaPod system. It also represents the first comprehensive validation of the GammaPod TPS, which is shown to be able to accurately calculate delivered dose distributions.

## CONFLICTS OF INTEREST

Ying Niu is a former employee of Xcision Medical Systems, LLC.
